# CircKIF5B Promotes Hepatocellular Carcinoma Progression by Regulating the miR-192 Family/XIAP Axis

**DOI:** 10.3389/fonc.2022.916246

**Published:** 2022-06-30

**Authors:** Zhenghua Fei, Yanfen Wang, Yuyang Gu, Rongrong Xie, Qiongyu Hao, Yiyan Jiang

**Affiliations:** ^1^ Department of Radiotherapy, The First Affiliated Hospital of Wenzhou Medical University, Wenzhou, China; ^2^ Department of Pathology, The Sixth Affiliated Hospital of Guangzhou Medical University, Qingyuan, China; ^3^ Division of Cancer Research and Training, Department of Internal Medicine, Charles Drew University of Medicine and Science, Los Angeles, CA, United States; ^4^ Department of Medical Oncology, The First Affiliated Hospital of Wenzhou Medical University, Wenzhou, China

**Keywords:** hepatocellular carcinoma, CircRNAs, microRNA, miR-192, XIAP

## Abstract

**Background:**

The long-term prognosis of HCC (hepatocellular carcinoma) with metastasis remains extremely poor. CircRNAs are promising as critical biological markers in identifying disease mechanisms and developing new effective treatments. However, the role of the aberrant expression of circRNAs in HCC progression remains largely unknown.

**Methods:**

CircKIF5B location was investigated by RNA fluorescence *in situ* hybridization (RNA-FISH). For circRNA determination, RNase R treatment and Real-Time Quantitative RT-PCR (qRT-PCR) were performed. Transwell chamber assays examined the chemotactic migration and invasion of liver cancer cells.

**Results:**

This study identified the circRNA circKIF5B originating from exons 1, 2, and 3 of the KIF5B gene. Importantly, we found that circKIF5B circRNA, rather than KIF5B linear mRNA, was notably upregulated in liver cancer cell lines and tissues. Moreover, we found that silencing circKIF5B markedly reduced the proliferation, invasion, and metastasis of liver cancer cells by sponging the miR-192 family, thus decreasing the expression of X-linked inhibitor of apoptosis (XIAP).

**Conclusion:**

Our data demonstrate that circKIF5B can regulate XIAP expression by sponging miR-192 and miR-215 competing for the ceRNA mechanism, indicating that circKIF5B may act as an essential upstream regulator and providing mechanistic evidence to support the view that circKIF5B/miR-192s/XIAP is a promising therapeutic target for treating liver cancer.

## Introduction

Primary liver cancer is the second leading cause of cancer-related death worldwide (1). Liver cancer comprises heterogeneous malignant tumors with different histological features that range from hepatocellular carcinoma (HCC) and intrahepatic cholangiocarcinoma (iCCA) to mixed hepatocellular cholangiocarcinoma (HCCCCA), fibrolamellar HCC (FLC), and the pediatric neoplasm hepatoblastoma (2). The long-term prognosis of HCC (hepatocellular carcinoma) with metastasis remains extremely poor (3). Genetic alterations in liver cancer cells result in hepatocellular heterogeneity, promoting liver cancer cell invasion and colonization in some organs during the metastatic process (4). Thus, there is an urgent need to better understand the underlying molecular mechanisms of HCC metastasis and identify novel therapeutic targets to improve clinical outcomes.

Circular RNAs (circRNAs) are a class of non-coding RNAs with a covalently closed loop structure that lacks 5’-3’ polarity or a polyadenylated tail (5). In the past decades, circRNAs have been identified in eukaryotic cells by electron microscopy and were previously considered as splicing error byproducts (6, 7). With the development of deep RNA sequencing, many exonic and intronic circRNAs have been identified to be expressed in multiple cell lines and various species (8–10), indicating that circRNAs are not simply byproducts of splicing errors. Unlike linear RNAs, most circRNAs are formed by exon or intron back-splicing. Recent studies of circRNA biogenesis have revealed that some circRNAs play essential roles in cancer development (11, 12). Some studies have reported that circRNAs function as “miRNA sponges” by modulating their activity on other target genes, which play an inhibitory role in miRNA regulation (13–16). Therefore, circRNAs are very promising as critical biological markers and precise diagnostics in identifying disease mechanisms and developing new methods for effective treatment. However, the role of aberrant expression of circRNAs in HCC progression remains largely unknown. This study identified and characterized the circRNA circKIF5B originating from exons 1, 2, and 3 of the KIF5B gene (10). Importantly, we found that circKIF5B circRNA, rather than KIF5B linear mRNA, was significantly upregulated in liver cancer tissues and cell lines. Furthermore, we found that inhibiting circKIF5B expression significantly reduced the invasion, metastasis, and proliferation of liver cancer cells *via* sponging of the miR-192 family, which downregulated the expression of X-linked inhibitor of apoptosis (XIAP).

## Materials & Methods

### Clinical Data/Patient Tissues

All procedures were carried out following guidelines set forth by the Declaration of Helsinki. Fresh tumor tissues were validated by pathological diagnosis, frozen in liquid nitrogen, and stored at −80°C. The clinicopathological data for 421 cases of liver cancer patients, as well as the relative gene expression levels (Hsa-miR-192, Hsa-miR-194, Hsa-miR-215, and XIAP), were downloaded from The Cancer Genome Atlas (TCGA) RNA-seq database (https://xenabrowser.net/).

### Cell Culture

Human embryonic kidney HEK293T (CRL-11268) cells, liver cancer cell lines HepG2, SK-HEP-1, SNU-475, SNU-449, PLC/PRF/5, SNU-387, SNU-423, and the normal liver cell line THLE-3 were initially obtained from the American Type Culture Collection (Rockville, MD, USA). Cells were cultured in DMEM (Dulbecco’s Modified Eagle’s Medium) or RPMI 1640 medium (Gibco, Carlsbad, CA, USA) according to the ATCC recommendation, supplemented with streptomycin (1,000 µg/ml), penicillin (1,000 units/ml), and 10% Fetal Bovine Serum (FBS) (Life Technologies, Grand Island, NY, USA) in an incubator with a humidified atmosphere of 5% CO_2_ at 37°C.

### Identification of circKIF5B Specific Binding With miRNAs

The circKIF5B-specific binding with miRNAs (Hsa-miR-192, Hsa-miR-194, and Hsa-miR-215) was identified using the Circular RNA Interactome database (https://circinteractome.nia.nih.gov/) in liver cancer cells. The target genes of Hsa-miR-192, Hsa-miR-194, and Hsa-miR-215 were screened using TargetScanHuman (http://www.targetscan.org/vert_72/) and miRDB (http://www.mirdb.org/).

### RNase R Treatment and Real-Time Quantitative RT-PCR (qRT-PCR)

Treatment with RNase R (3 U/mg) (Biosearch Technologies, Middlesex, UK) was used to degrade linear mRNA for circRNA detection. In brief, total RNA was isolated with the RNeasy Mini Kit (QIAGEN, Stockach, Germany). The total RNA was incubated with RNase R at 37°C for 1 h. The cDNA was synthesized using an iScript™ reverse transcription kit (Bio-Rad, Hercules, CA, USA). Circular RNA was quantified using a Universal SYBR^®^ Green Supermix (Bio-Rad, Hercules, CA, USA). The real-time PCR was conducted using the CFX96 Real-Time PCR Detection System (Bio-Rad, Hercules, CA, USA). Human 18S was selected as a housekeeping gene for mRNA and circRNA. The expression of miRNA was normalized to hsa-miR-26a-5p (477995_mir). TaqMan miRNA assays (Thermofisher Scientific, Waltham, MA, USA) were used to perform qRT-PCR for miRNA detection. The relative gene expression of circRNA and mRNA was normalized to 18S and determined by the 2^−[ΔCT (sample) − ΔCT (calibrator)]^ method. For all primers see **Table S1**.

### Western Blot Analysis

Total proteins from the cells were extracted using a RIPA Lysis and Extraction Buffer (Thermo Scientific™, Waltham, MA, USA) including Halt™ Protease and Phosphatase Inhibitor Cocktail (100×) (Thermo Scientific™, Waltham, MA, USA). Proteins were separated by sodium dodecyl sulfate-polyacrylamide gel electrophoresis (SDS-PAGE) and then transferred to polyvinylidene difluoride (PVDF) membranes (Millipore, Burlington, MA, USA). The PVDF membranes were incubated with primary antibodies at 4°C overnight, and then further incubated with secondary antibodies for 30 min on the following day. The immunoreactive signals were visualized using the chemiluminescence reagents WesternSure^®^ PREMIUM Chemiluminescent Substrate (LI-COR^®^, Lincoln, NE, USA). Images were captured with the Odyssey^®^ XF Imaging System (LI-COR Biosciences, Lincoln, NE, USA). The following antibodies were used: XIAP Antibody (Cell Signaling Technology, Danvers, MA, USA), α-Tubulin Antibody (Cell Signaling Technology, Danvers, MA, USA).

### CircRNA *In Vivo* Precipitation (circRIP) and Biotin-Labeled miRNA Capture

HepG2 and SK-HEP-1 cells were transfected with the specific biotin-tagged circKIF5B probe (**Table S1**), biotin-labeled miR-192, and miR-215 mimics at a final concentration of 200 nmol/L for 24 h. The cells were fixed with 1% formaldehyde for 10 min when they reached sufficient confluency. Then the cells were lysed and sonicated, and then incubated with the streptavidin-dynabead mixture (Invitrogen, Waltham, MA, USA) overnight at 4°C. The mixture was washed with lysis buffer containing proteinase K. The abundance of circKIF5B and miR-192/miR-215 in the binding fractions was tested by qRT-PCR.

### RNA Fluorescence *In Situ* Hybridization (RNA-FISH)

A fluorescent *in situ* hybridization kit (Stellaris RNA FISH Hybridization) was used to study the location of circKIF5B in accordance with the instructions of the manufacturer. For Digoxin (DIG)-labeled sense or antisense probes for the junction sequence of circKIF5B, see **Table S1**. Adherent cells were fixed on an 18 mm cover glass in a 6-well cell culture plate. Wash twice with 1 ml of 1× PBS. Permeabilize cells in 1 ml of 70% (vol/vol) ethanol for at least 1 hour at 4°C. Hybridization in adherent cells: 1 µl of probe stock solution was added to 100 µl of hybridization buffer, vortexed, and centrifuged to prepare the buffer containing the probe. Incubate the cells with hybridization buffer in the dark at 37°C for at least 4 h. DAPI nuclear stain was used to counter-stain the nuclei. A small drop of Vectashield Mounting Medium was added onto a microscope slide, and then coverglass was mounted onto the slide. The coverglass perimeter was sealed with a clear nail. Proceed to the image after polishing and drying.

### Luciferase Reporter Assays

The WT fragment of the XIAP 3’-UTR and the mutation fragment at the miR-192-5p binding sites were cloned into psiCHECK-2 vectors, respectively. For reporter assays, WT or mutant reporter vector, psiCHECK-2-XIAP 3’-UTR: WT or MUT, was co-transfected with hsa-miR-192, hsa-miR-194, and hsa-miR-215 mimics, respectively, into HepG2 and SK-HEP-1 cells. Twenty-four hours after transfection, cell lysate was subjected to luciferase activity assessment using the Dual-Glo system (Promega Corp, WI, USA).

### Xenograft Nude Mouse Model

All mice studies followed the principles and procedures in the Guide for the Care and Use of Animals of The First Affiliated Hospital of Wenzhou Medical University. The mice were maintained in HEPA-filtered racks in a pathogen-free barrier facility and fed an autoclaved laboratory rodent diet. To establish the subcutaneous xenograft *in vivo* model, ~5 × 10^6^ logarithmically growing CircKIF5B-overexpressing HepG2 cells or control parental cells in 0.1 ml Matrigel Matrix (Corning, Cat#: CB-40234) were subcutaneously injected into the left or right flank of 3-week-old male BALB/c nu/nu mice (n = 6). Mice were sacrificed, and tumor tissues were excised and weighed about 20 days later. The tumor volume was calculated using the equation: volume = width^2^ ×length × 0.5 (mm^3^).

### Cell Proliferation

The cell counting kit-8 (CCK8) (MilliporeSigma, Burlington, MA, USA) was used to assess cell proliferation. Briefly, the cells were seeded in a 96-well plate at a density of ~5,000 per well. Cell viability was assessed 48–56 h later. The culture medium was replaced with 100 μl of complete medium containing 10 μl of CCK8, and the cells were incubated in an incubator for another 2 h. The optical density (O.D.) was measured at 450 nm in a GloMax^®^-Multi+ Detection System (Promega Corp, WI, USA).

### Cell Migration and Invasion Activity

The chemotactic migration and invasion activity of HepG2 and SK-HEP-1 cells were examined with the Transwell Chamber Assay. Cells were seeded in a QCM 24-well Migration Assay plate (Sigma-Aldrich. Cat#: ECM508) and a QCM Collagen Cell 24-well Invasion Assay plate (Sigma-Aldrich. Cat#: ECM551) with an 8 µm pore size and colorimetric detection. The serum-free or complete RPMI 1640 medium was added to the upper inserts or bottom chamber, respectively. After culture for 24–36 h, non-migrating/invading cells on the membrane were removed by a swab, and migrated/invaded cells underneath the membrane were stained with crystal violet staining solutions according to the instructions of the manufacturer. The cell number was counted on the lower side of the filter under a microscope.

### Immunohistochemical Staining and Scoring

Briefly, the tissue array specimens embedded in paraffin slices on coated slides were washed in xylene to remove the paraffin, rehydrated through serial dilutions of alcohol, followed by washings with a solution of PBS. Treated sections were then placed in a citrate buffer (pH 6.0) and heated in a microwave for 5 min. The samples were then incubated with an antibody for 60 min at RT. The conventional streptavidin peroxidase method was performed for signal development, and the cells were counter-stained with hematoxylin. This slide was mounted with gum for examination and capture by the MoticEasyScan One system. Every tumor was given a score according to the intensity of the nuclear or cytoplasmic staining (no staining = 0; weak staining = 1; moderate staining = 2; strong staining = 3) and the extent of stained cells (0% = 0; 1–10% = 1; 11–50% = 2; 51–80% = 3; 81–100% = 4). The final immunoreactive score was determined by multiplying the intensity and extent of positivity scores of stained cells, with a minimum score of 0 and a maximum score of 12 (17).

### Statistical Analysis

The data were expressed as the mean ± standard deviation (SD) of at least three independent experiments and were analyzed with Prism 6.0 software (GraphPad, San Diego, CA, USA). The Student’s t-test was used to analyze the statistical significance between the two groups, while multiple-group comparison was performed with one-way analysis of variance (ANOVA). P-values less than 0.05 were considered statistically significant. Univariate analysis and multivariate logistic regression investigated the association between patient characteristics and circRNA expression.

## Results

### CircKIF5B Is Correlated With Poor Clinical Outcomes of Liver Cancer Patients

**Figure 1:**
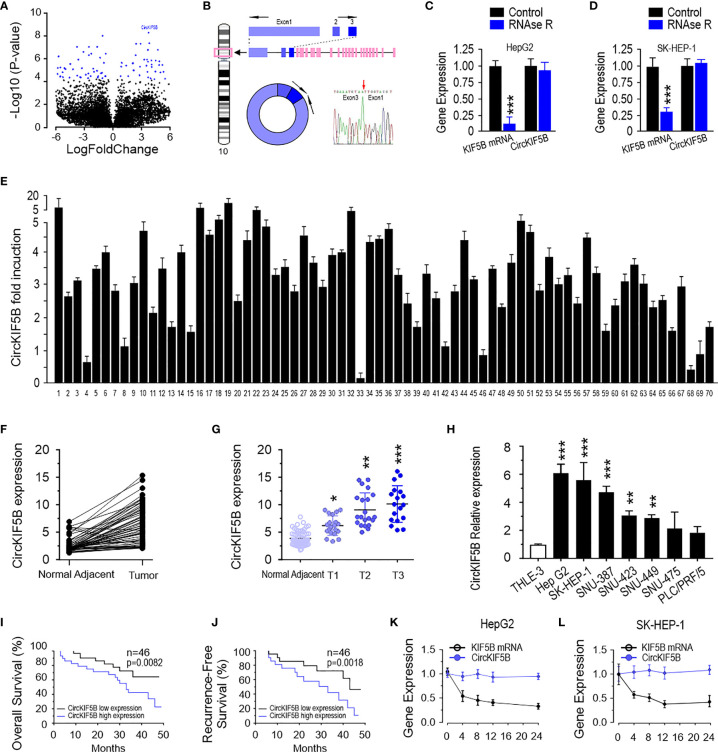
CircKIF5B is up-regulated in liver cancer tissue. **(A)** A volcano plot for fold changes of circRNAs level in liver cancer tissues compared adjacent normal tissues. **(B)** Schematic illustration indicating circKIF5B formation by the circularization of KIF5B exons 1, 2, and 3. **(C)** CircKIF5B and KIF5B transcripts levels in the presence or absence of RNase R treatment by qRT-PCR in HepG2 and SK-HEP-1 **(D)** cells. **(E)** The relative expression of circKIF5B by qRT-PCR analysis with divergent primers in liver cancer tissues compared with adjacent normal tissues in 70 pairs of liver cancer patients. **(F)** The expression of circKIF5B in 70 pairs of human samples **(E)** with the Mann–Whitney test. **(G)** Correlation between circKIF5B level and the stage of clinical classification in 70 cases of liver cancer and noncancerous tissues. **(H)** The circKIF5B level in liver cancer cell lines and normal liver cell line. **(I, J)** The curves of overall survival **(I)** and recurrence-free survival **(J)** association with circKIF5B level in 46 liver cancer patients. **(K, L)** CircKIF5B and linear KIF5B transcript level after actinomycin D treatment. *p < 0.05, **p < 0.01, ***P < 0.005.

A circRNA microarray was performed to study the differential expression profile of circRNA in liver cancer tissues. The circRNA variation was shown in the volcano plot based on log_2_ (fold changes) ≥1, p <0.05 (**Figure 1A**). According to circBase (18), hsa_circ_0018105 has consisted of KIF5B exons 1–3, referred to as circKIF5B hereafter (**Figure 1B**). The results of Sanger sequencing showed that the sequence of the PCR product was derived from exons 1 to 3 of the KIF5B gene (**Figure 1B**), which was consistent with that in circBase. The RNase-R assay showed that circKIF5B was resistant to RNase-R in HepG2 and SK-HEP-1 cells (**Figures 1C, D**). The level of circKIF5B in 70 pairs of liver cancer tissues was determined by qPCR. The results demonstrated that circKIF5B had a significantly high level in liver cancer tissues (**Figure 1E**). Specifically, paired analysis of liver cancer samples indicated that the circKIF5B was higher in most patient samples (**Figure 1F**). The circKIF5B level was also associated with the histological grade and pathological stage (**Figure 1G**). In addition, the circKIF5B level is higher in 6 liver cancer cell lines (HepG2, SK-HEP-1, SNU-387, SNU-423, SNU-449, SNU-475, and PLC/PRF/5) compared to THLE-3, which is a normal liver cell line (**Figure 1H**). The association of circKIF5B and the prognosis of liver cancer patients was further analyzed in the 46 patients (**Figures 1I, J**). In all cases, the median value of the circKIF5B level was chosen as the cutoff value to separate the dataset into high and low circKIF5B expression. The Kaplan–Meier analysis revealed a reverse association between the circKIF5B level and the overall survival (OS) of patients or the recurrence-free survival (RFS) time. Furthermore, after being treated with actinomycin D, linear KIF5B was significantly decreased, while circKIF5B expression was not in HepG2 and SK-HEP-1 cells (**Figures 1K, L**).

### Knockdown of circKIF5B Inhibits Proliferation and Metastasis of Liver Cancer Cells

**Figure 2:**
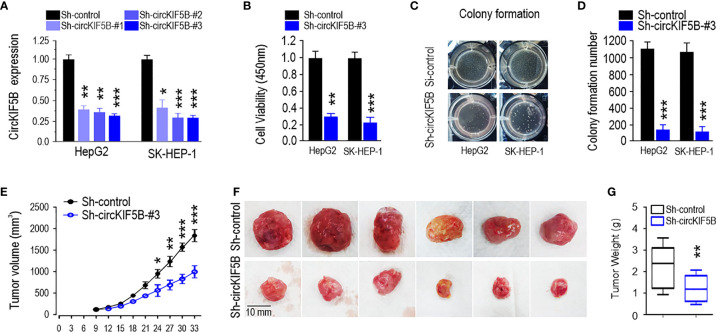
CircKIF5B knockdown inhibits growth and metastasis of HCC cells. **(A)** Knockdown efficiency of si-circKIF5B in HepG2 and SK-HEP-1 cells. **(B)** Cell viability by CCK-8 assay in HepG2 and SK-HEP-1 cells. **(C)** Colony-forming ability in HepG2 and SK-HEP-1 cells. **(D)** Quantification of panel (**C**). **(E)** Xenograft assay with HepG2 stable cell lines. **(F)** Knockdown of circKIF5B decreased the volume of the HepG2 xenograft tumors. **(G)** Knockdown of circKIF5B decreased the weight of the HepG2 xenograft tumors. *p < 0.05, **p < 0.01, ***P < 0.005.

A stable circKIF5B knockdown cell line was successfully established by transducing circKIF5B knockdown lentiviral particles into HepG2 and SK-HEP-1 cells (**Figure 2A**). The CCK8 assay showed that the cell growth ability was weakened in sh-circKIF5B transduced HCC cells (**Figure 2B**). As shown in **Figure 2C**, results of colony formation demonstrated that the silencing of circKIF5B inhibited the proliferative capacity of HepG2 and SK-HEP-1 cells (**Figure 2D**, quantification of **Figure 2C**). HepG2 cells that stably silence circKIF5B were injected into the subcutaneous tissues of nude mice, the volumes and weight of the tumors derived by circKIF5B knockdown HCC cells grew slower than those formed by control cells (**Figures 2E–G**). These results reveal that silencing of circKIF5B inhibited subcutaneous xenograft growth.

### Knockdown of circKIF5B Inhibits Liver Cancer Cell Migration and Invasion and Tumor Metastasis

**Figure 3:**
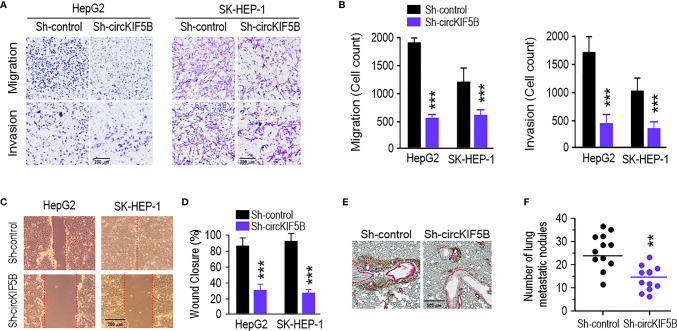
CircKIF5B knockdown depresses HCC cells migration and invasion and tumor metastasis. **(A)** Repressing circKIF5B suppressed the migration and invasion of HepG2 and SK-HEP-1 cells transfected with control or circKIF5B siRNAs. **(B)** Colorimetric measurement of panel **(A)**. **(C)** The effect of si-circKIF5B on cell migration evaluated by wound healing assay in HepG2 and SK-HEP-1 cells. **(D)** Quantification of panel **(C)**. **(E)** Lung metastatic colonies in nude mice injected with HepG2 cells stably transfected with si-circKIF5B or control si-RNA. **(F)** Quantification of panel **(E)**. *p < 0.05, **p < 0.01, ***P < 0.005.

To investigate the correlation between circKIF5B and the metastatic ability of HCC cells, transwell assays were used to assess the effect on the migration and invasion of HCC cells *in vitro*. As expected, the migration and invasion capacities of the HCC cells were remarkably weakened by circKIF5B knockdown (**Figures 3A, B**). The wound-healing assay also indicated that circKIF5B silencing notably blocked HCC cell migration (**Figures 3C, D**). Subsequently, ectopic knockdown of circKIF5B inhibited the potential of HCC cells to metastasize to the lungs, which was confirmed by an *in vivo* tail vein injection model (**Figures 3E, F**).

### CircKIF5B Abundantly Sponges the miR-192 Family in HCC Cells

**Figure 4:**
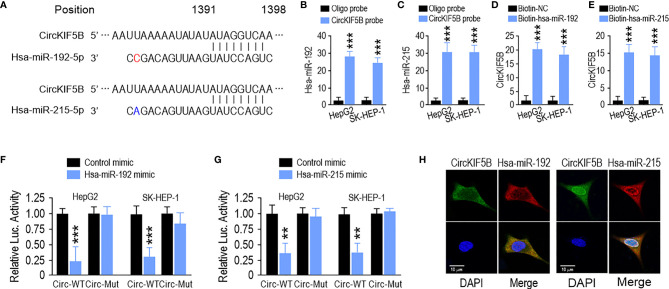
CircKIF5B functions as a sponge for the miR-192 family in HCC cells. **(A)** The predicted binding sites of miR-192 and miR-215 within the CircKIF5B. **(B,C)** The relative levels of miR-192 and miR-215 in the HepG2 and SK-HEP-1 cell lysates were detected by qRT-PCR. miR-192 and miR-215 were pulled down by circKIF5B probe in HepG2 and SK-HEP-1 cells. **(D,E)** CircKIF5B levels were quantified after the capture of a biotinylated miR-192 probe by qRT-PCR in HepG2 and SK-HEP-1 cells. **(F,G)** Luciferase reporter activity confirmed the interaction between miR-192s and circKIF5B. **(H)** RNA FISH for circKIF5B and miR-192 detection in HepG2 cells. Blue (DAPI) represented nuclei, green represented circKIF5B, and red represented miR-192 or miR-215. *p < 0.05, **p < 0.01, ***P < 0.005.

Next, we focused on the capability of circKIF5B to bind to miRNAs. The miR-192 family was the best possible target of circKIF5B based on the prediction from the Circular RNA Interactome (https://circinteractome.nia.nih.gov/) (**Figure 4A**). RNA immunoprecipitation (RIP) data demonstrated significant enrichment of miR-192 and miR-215 on circKIF5B by a biotin-labeled circKIF5B probe (**Figures 4B,C**), showing that miR-192 and miR-215 were sponged by circKIF5B in HepG2 and SK-HEP-1 cells. Reciprocally, circKIF5B was also enriched in both miR-192 and miR-215 using a biotin-labeled miRNA pulldown in HCC cells (**Figures 4D, E**). However, miR-194, another member of the miR-192 family, and circKIF5B were not enriched reciprocally with a biotin-labeled pulldown assay (**Figures S1A, B**). A luciferase reporter (Luc_circKIF5B) was constructed by inserting the circKIF5B fragment into the luciferase gene downstream. The MiR-192 family of mimics and reporter genes were co-transfected into HepG2 and SK-HEP-1 cells. A notable reduction in luciferase reporter activity was observed when miR-192 or miR-215 mimics were co-transfected with wild-type luciferase reporter (**Figures 4F, G**). Co-transfection with mutant luciferase reporter and miR-192 family mimics did not change the luciferase reporter activity in HepG2 and SK-HEP-1 cells (**Figures 4F, G**). MiR-194 also did not change the luciferase reporter activity of Luc_circKIF5B (**Figure S1C**). Furthermore, the RNA FISH assay indicating that circKIF5B co-localized with miR-192 or miR-215 in the cytoplasm of HepG2 cells (**Figure 4H**) supported previous interaction findings. These findings suggest that circKIF5B acts as a sponge for the miR-192 family.

### miR-192 Family Inhibits Liver Cancer Progression by Targeting XIAP

**Figure 5:**
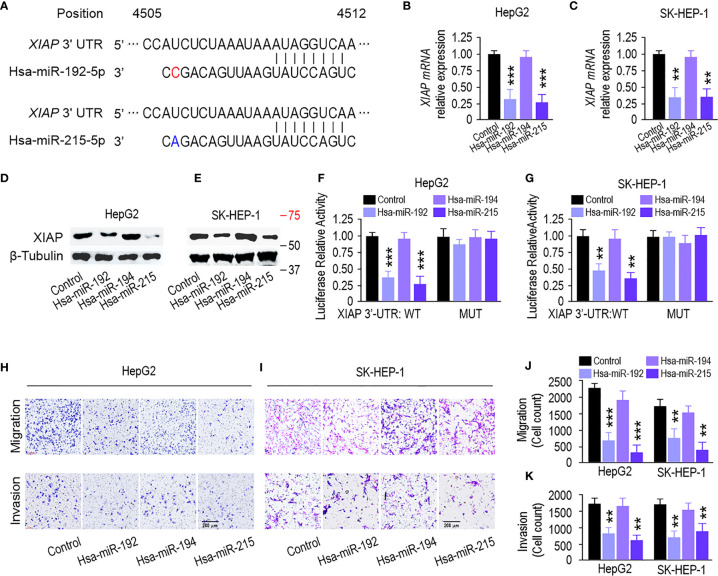
miR-192 family inhibits liver cancer progression through targeting XIAP. **(A)** Predicted miR-192s binding sites in XIAP 3’-UTR by bioinformatics analysis. **(B, C)** miR-192s decreased the mRNA level and protein expression **(D, E)** of XIAP in HepG2 and SK-HEP-1 cells. **(F, G)** Luciferase reporter activity confirmed the interaction between miR-192s and XIAP in HepG2 and SK-HEP-1 cells. **(H, I)** Migration and invasion capabilities evaluated by transwell assays in HepG2 and SK-HEP-1 cells transfected with miR-192s mimics. **(J, K)** Colorimetric measurement of panels **(H, I)**. (*p < 0.05, **p < 0.01, ***P < 0.005).

The three prime untranslated regions (3′-UTR) of XIAP harbor both miR-192 and miR-215 binding sites based on the algorithm from miRanda (http://mirdb.org/) and TargetScan (http://www.targetscan.org/) (**Figure 5A**). qPCR and immunoblotting analysis showed that miR-192 and miR-215 mimics could inhibit XIAP at the mRNA and protein levels in HepG2 and SK-HEP-1 cells (**Figures 5B-E**). However, miR-194 did not change the XIAP level (**Figures 5B–E**). Next, to verify this interaction, luciferase reporter assays showed that transfection of miR-192 and miR-215 mimics significantly inhibited the activity of a luciferase reporter carrying the wild-type XIAP 3′-UTR. However, the activity of the luciferase reporter taking the mutated XIAP 3′-UTR was not changed by miR-192 or miR-215 (**Figures 5F, G**). Transwell assays showed that overexpression of miR-192 and miR-215, but not miR-194, markedly suppressed migration and invasion in HepG2 and SK-HEP-1 cells (**Figures 5H–K**). Furthermore, overexpression of XIAP rescued miR-192, causing migration and invasion suppression in HepG2 and SK-HEP-1 cells (**Figures S2A, B**). These results suggest that miR-192 and miR-215 regulate the migration and invasion of HCC cells by targeting XIAP.

### CircKIF5B Acts as a ceRNA to Regulate XIAP

**Figure 6:**
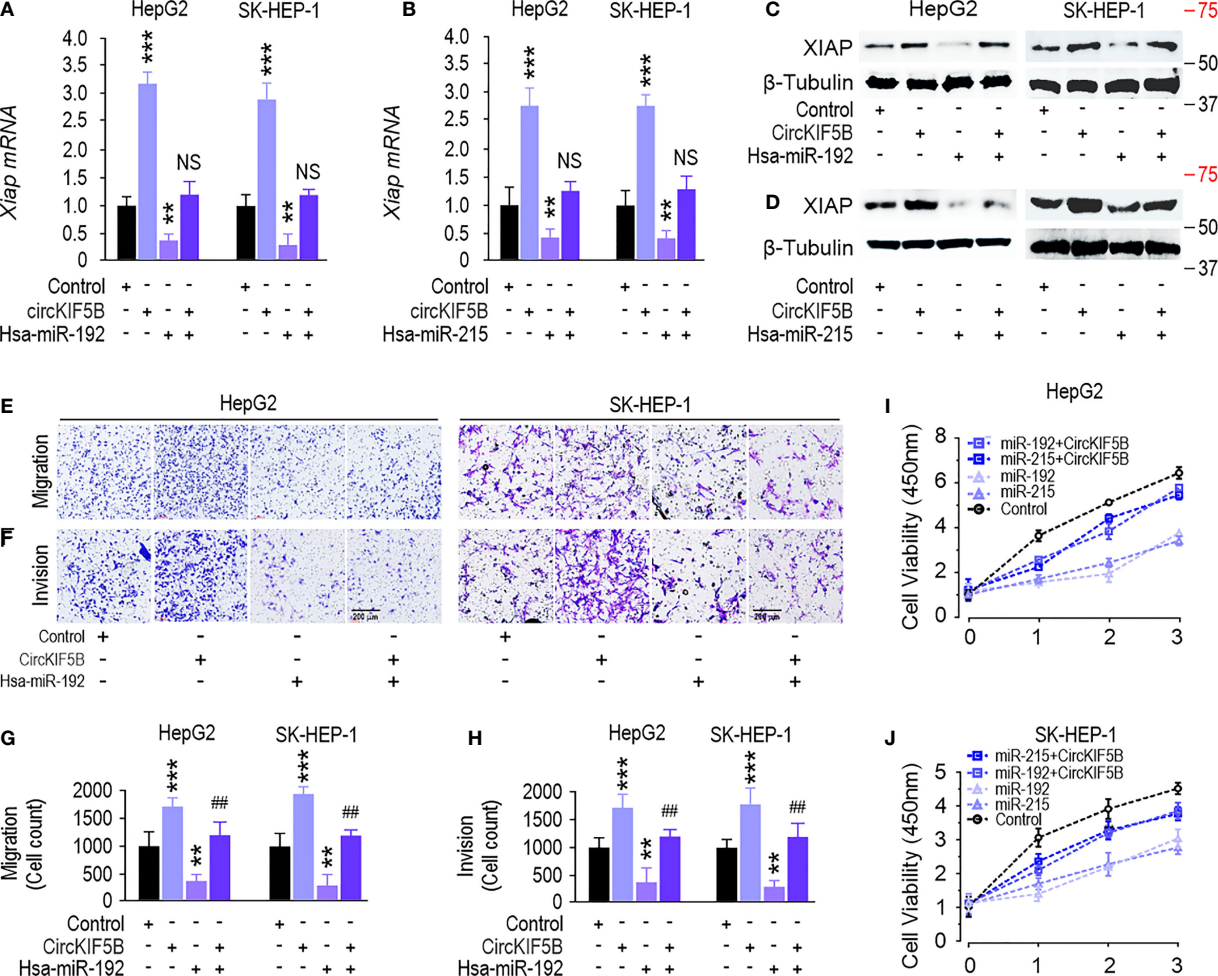
CircKIF5B regulates XIAP expression and inhibits liver cancer progression *via* targeting the miR-192 family. **(A, B)** qRT-PCR showed that miR-192 family attenuated circKIF5B caused XIAP mRNA expression increase. **(C, D)** Western blot showed that miR-192 family attenuated circKIF5B caused XIAP protein expression increase. **(E, F)** Migration and invasion ability of circKIF5B and miR-192s in HepG2 and SK-HEP-1 cells. **(G, H)** Colorimetric measurement of panels **(E, F)**. **(I, J)** The viability of circKIF5B and miR-192s evaluated by CCK-8 assay in HepG2 and SK-HEP-1 cells. *p < 0.05, **p < 0.01, ***P < 0.005; ^##^p < 0.01 compared with miR-192 group.

CircKIF5B overexpression increased XIAP expression at the mRNA and protein levels (**Figures 6A–D**). Simultaneously, transfection with a miR-192 or miR-215 mimic reversed this increase in HepG2 and SK-HEP-1 cells (**Figures 6A–D**), which indicates that circKIF5B sponges miR-192 and miR-215 to regulate XIAP expression in HCC cells. Furthermore, transfection of miR-192 mimic significantly suppressed the migration and invasion, while overexpression of circKIF5B rescued miR-192 caused migration and invasion suppression in HepG2 and SK-HEP-1 cells (**Figures 6E–H**). CCK-8 assay showed that miR-192 or miR-215 significantly inhibited HepG2 and SK-HEP-1 cell proliferation, while circKIF5B rescued miR-192 or miR-215 and led to proliferation inhibition of HepG2 and SK-HEP-1 cells (**Figures 6I, J**). Transwell assays showed that overexpression of miR-192 and miR-215, but not miR-194, significantly suppressed the migration and invasion of HepG2 and SK-HEP-1 cells (**Figures 5H–K**). A luciferase reporter assay demonstrated that overexpression of miR-192 or miR-215 attenuated circKIF5B increased luciferase activity of XIAP 3′-UTR in HepG2 and SK-HEP-1 cells (**Figures S3A, B**). These findings reveal that circKIF5B promotes HCC cell growth by sponging miR-192 and miR-215.

### CircKIF5B Promotes Tumorigenesis *In Vivo* Through XIAP Signaling

**Figure 7:**
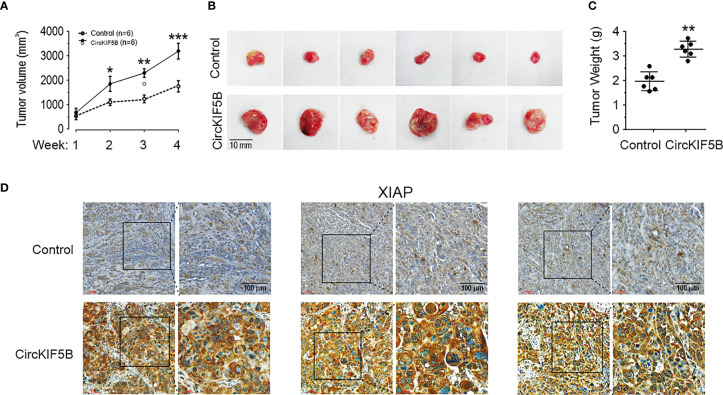
CircKIF5B promotes tumors growth through XIAP signaling. **(A)** Xenograft assay with HepG2 stable cell lines. Six nude mice were used in each group. **(B)** Overexpression of circKIF5B increased the volume of the xenograft tumors. **(C)** Overexpression of circKIF5B increased the weight of the xenograft tumors. **(D)** IHC staining of xenograft tumor tissues using the XIAP antibodies. *p < 0.05, **p < 0.01, ***P < 0.005.

To evaluate the regulation mechanisms between circKIF5B and XIAP *in vivo*, tumor growth was examined by subcutaneous injection of circKIF5B overexpressed HepG2 cells into nude mice. Cells overexpressing circKIF5B had a higher growth rate than the control (**Figures 7A, B**). Consistent data for the average tumor wet weight was observed (**Figure 7C**). Upregulated circKIF5B level increased the mean immunopositive area for XIAP in tumor tissues, as determined by immunohistochemistry (IHC) (**Figure 7D**).

### Elevated circKIF5B and Decreased miR-192 Family Expression Contribute to Liver Cancer progression

**Figure 8:**
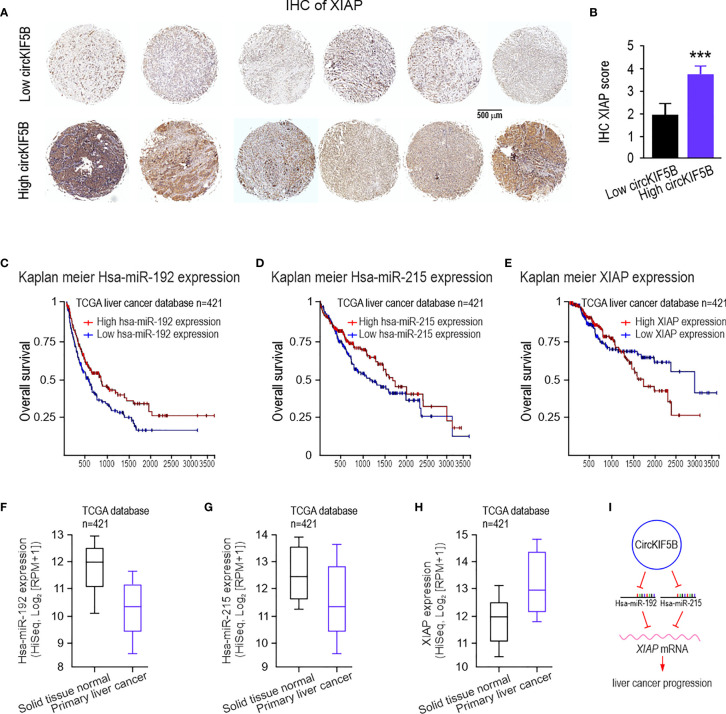
The clinical significance of circKIF5B, miR-192s, and XIAP in human liver cancer patients. **(A)** IHC staining for liver cancer tissue microarrays (TMAs) with liver cancer and adjacent normal liver tissues using the XIAP antibodies. **(B)** IHC XIAP staining score analysis for TMAs. **(C–E)** The overall survival curves of patients with liver cancer stratified by miR-192s and XIAP levels in their primary tumors. **(F–H)** RNA-seq data for expression of the miR-192 family **(F, G)** and XIAP **(H)**, in solid tissue normal (n = 50) and primary tumor (n = 371) in the TCGA database. **(I)** Function and mechanism of circKIF5B during liver cancer progression. ***P < 0.005.

To evaluate the clinical relevance between circKIF5B and XIAP in liver cancer progression, XIAP expression was determined by IHC staining of XIAP antibody from 70 liver cancer samples (**Figure 8A**). Based on the median value, the 70 liver cancer cases were divided into low- and high-circKIF5B level groups. The results showed that higher expression of circKIF5B was significantly correlated with a higher XIAP level (**Figure 8B**). Furthermore, Kaplan–Meier survival analysis revealed that high XIAP expression or low miR-192s levels are associated with poorer overall survival (**Figures 8C–E**) and recurrence-free survival rates of liver cancer patients (**Figures S4C–E**). RNA-seq data analysis based on TCGA Liver databases (n = 421) by XENA revealed that miR-192 and miR-215 levels decreased in primary liver cancer tissue compared with adjacent normal tissues (**Figures 8F, G**). However, XIAP is highly expressed in liver cancer patients (**Figure 8H**). Correlation analysis revealed that miR-192 or miR-215 is significantly negatively associated with the XIAP levels (**Figures S4A, B**). Taken together, the high expression of circKIF5B in liver cancer tissue and the trends of high expression related to low survival rates demonstrate that circKIF5B is a proto-oncogene sponge miR-192 family to mediate liver cancer progression (**Figure 8I**).

## Discussion

With the advent of next-generation sequencing technologies (12, 19, 20) and the development of bioinformatics, numerous circular RNAs have been identified and studied, spanning various species. Many highly stable circRNAs are abundantly expressed, and circRNA research has been a hot topic in many diseases, especially in malignant tumor research (21–23). However, although studies on circRNAs are being developed rapidly, many questions are still unanswered. Increasing evidence shows that circRNAs are critical post-transcriptional regulators of the RNA regulatory network in the early stages of HCC (24–26).

Several studies have revealed that the KIF5B gene may play an essential role in lung cancer invasion and metastasis (27–29). We hypothesize that circRNAs may affect liver cancer progression with cell type and developmental stage-specific characteristics. Our study has shown that circKIF5B is highly expressed in HCC cell lines and tissues. As demonstrated by loss-of-function assays, circKIF5B silencing suppresses the tumor growth. CircRNAs have been characterized as a miRNA “sponge” to reduce the number of miRNAs as a vital factor in hepatocellular carcinoma (30, 31). Our findings also revealed that circKIF5B plays a regulatory role by harboring miR-192 family members, miR-192, and miR-215 (not including miR-194). MiRNA-192 and miR-215 have been associated with promoting or suppressing tumors in various types of solid tumors (32–36). miR-215 was found to suppress papillary thyroid cancer metastasis by regulating the EMT *via* the AKT/GSK-3β/Snail signaling (37). Moreover, Wang et al. identified that miR-192-3p directly targeted the 3′-UTR of XIAP, and this process was necessary for HBV replication *in vitro* and *in vivo* (38). However, the role of the miR-192 family in the pathogenesis of liver cancer is not clear. We demonstrated that miR-192 and miR-215 inhibited liver cancer cell growth *in vitro*. Intriguingly, we found that the miR-192 family directly targets XIAP, which stops viral infection and causes apoptotic cell death by binding to and inhibiting caspases 3, 7, and 9 (39). Most importantly, we revealed that circKIF5B plays an oncogenic role by sponging miR-192 and miR-215 to rescue the expression of XIAP, which is a protein that stops apoptotic cell death. However, there are some limitations to this study. To prove circKIF5B elicits HCC initiation by miR-192/XIAP, more apoptotic-related studies need to be investigated, which is the emphasis of our next investigation.

## Conclusions

Our findings reveal that circKIF5B can regulate XIAP expression by sponging miR-192 and miR-215 competing for the ceRNA mechanism, indicating that circKIF5B acts as a critical upstream regulator and supporting the notion that circKIF5B/miR-192s/XIAP is a promising therapeutic target for liver cancer. This study demonstrates that the potential role of circRNAs in cancer can served as valuable clinical prognostic biomarkers and therapeutic targets for surveillance and early treatment of HCC.

## Data Availability Statement

The raw data supporting the conclusions of this article will be made available by the authors, without undue reservation.

## Ethics Statement

The animal study was reviewed and approved by the Committee of Care and Use of Animals of The First Affiliated Hospital of Wenzhou Medical University.

## Author Contributions

Study concept and design: QH and YJ. Acquisition of data: ZF, YW, RX, YG, and QH. Analysis and interpretation of data: ZF and YJ. Drafting of the manuscript: ZF. Critical revision of the manuscript: YJ. All authors listed have made a substantial, direct, and intellectual contribution to the work and approved it for publication.

## Funding

This study was supported by a grant from the Natural Science Foundation of Zhejiang Province (No. LY21H280012) and the Science and Technology Bureau of Wenzhou [No. Y20190708]. This work was supported by the NIH/NIMHD Accelerating Excellence in Translational Science Pilot Grant G0814C01 (QH).

## Conflict of Interest

The authors declare that the research was conducted in the absence of any commercial or financial relationships that could be construed as a potential conflict of interest.

## Publisher’s Note

All claims expressed in this article are solely those of the authors and do not necessarily represent those of their affiliated organizations, or those of the publisher, the editors and the reviewers. Any product that may be evaluated in this article, or claim that may be made by its manufacturer, is not guaranteed or endorsed by the publisher.
