# Supplementary figures and images for: CircKIF5B Promotes Hepatocellular Carcinoma Progression by Regulating the miR-192 Family/XIAP Axis
Source: Front Oncol. 2022 Jun 30;12:916246. doi: 10.3389/fonc.2022.916246 (PMC9281474; doi:10.3389/fonc.2022.916246)

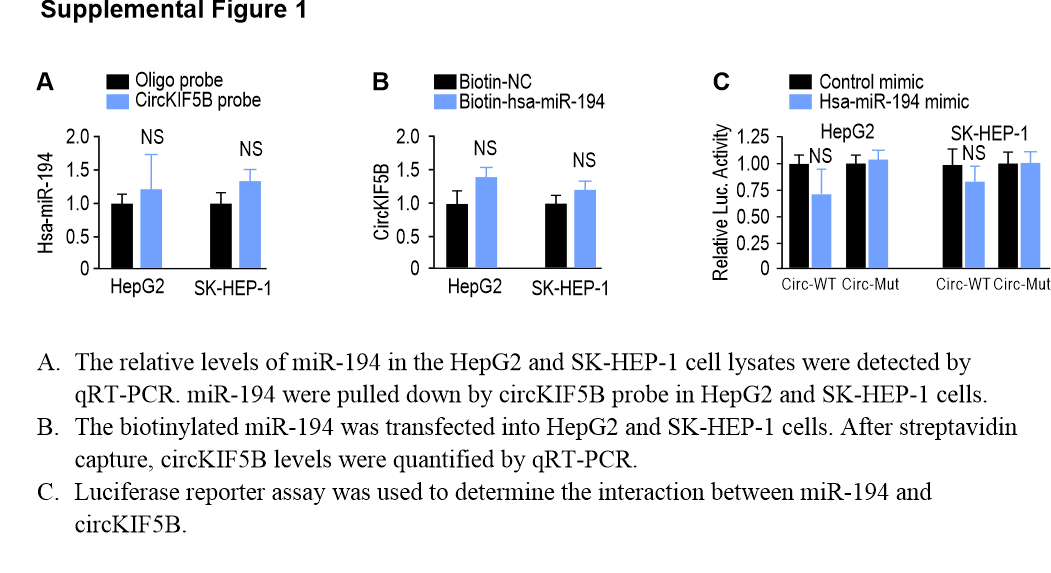

Supplement: Supplementary file 1 [file Image_1.tif]

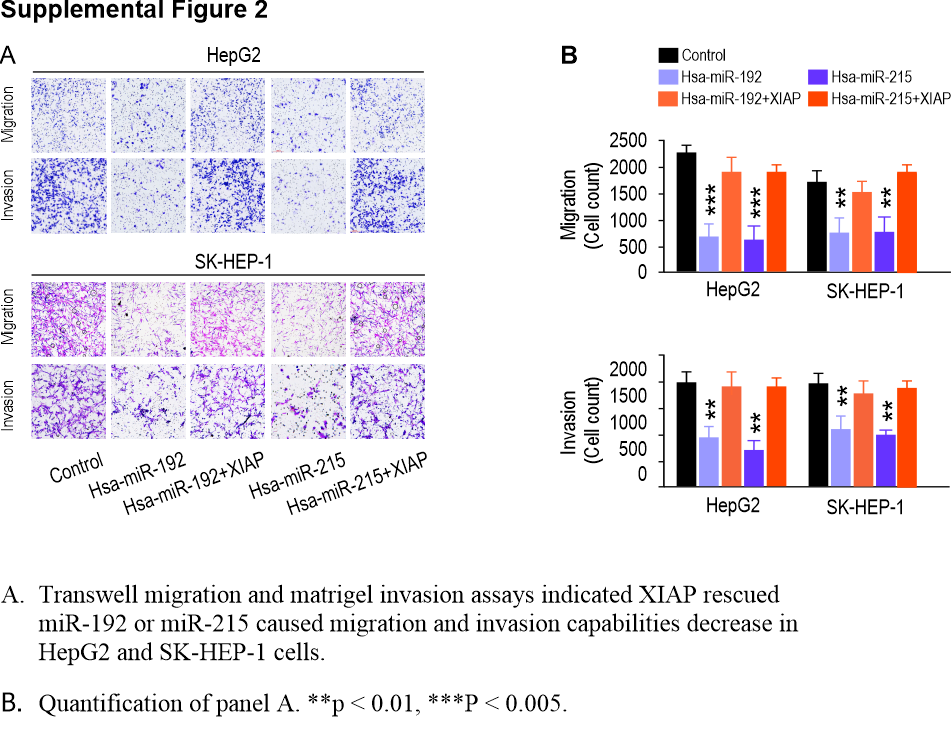

Supplement: Supplementary file 2 [file Image_2.tif]

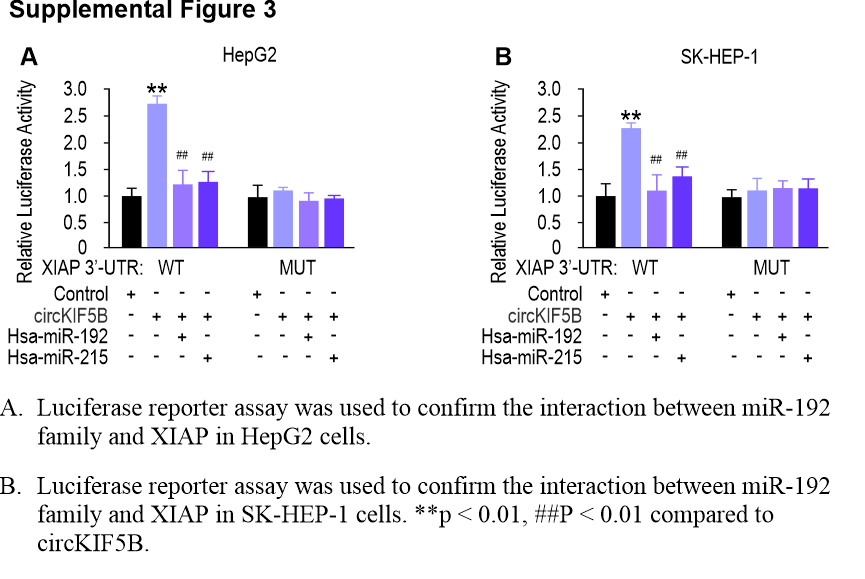

Supplement: Supplementary file 3 [file Image_3.tif]

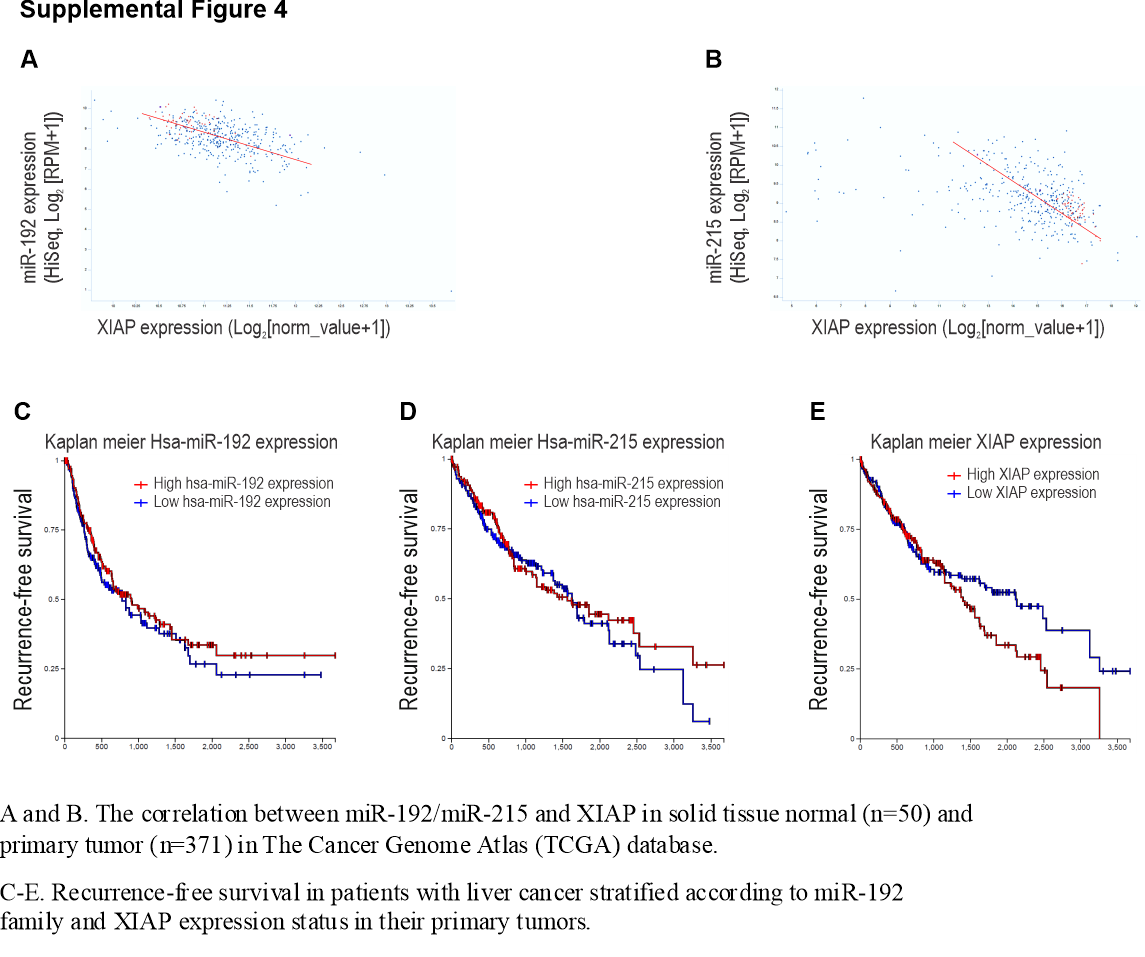

Supplement: Supplementary file 4 [file Image_4.tif]
