# Supplementary material for: CircKIF5B Promotes Hepatocellular Carcinoma Progression by Regulating the miR-192 Family/XIAP Axis
Source: Front Oncol. 2022 Jun 30;12:916246. doi: 10.3389/fonc.2022.916246 (PMC9281474; doi:10.3389/fonc.2022.916246)
Supplement: Supplementary file 5 [file Table_1.docx]

Table S1 Primers used for quantitative real-time PCR and probe for RNA FISH

Table S2 Primers used for lentivirus production and shRNAs sequences
